# Supplementary material for: Ultrathin organosiloxane membrane for precision organic solvent nanofiltration
Source: Nat Commun. 2024 Mar 30;15:2800. doi: 10.1038/s41467-024-47115-9 (PMC10981765; doi:10.1038/s41467-024-47115-9)
Supplement: Supplementary file 1 — Supplementary Information [file 41467_2024_47115_MOESM1_ESM.pdf]

Supplementary Information

Ultrathin Organosiloxane Membrane for Precision Organic Solvent Nanofiltration

Jihoon Choi[1,†], Keonwoo Choi[1,†], YongSung Kwon[2], Daehun Kim[1,2], Youngmin Yoo[2], Sung Gap Im[1,3,*], Dong-Yeun Koh[1,3,*]

Affiliations

[1]Department of Chemical and Biomolecular Engineering (BK21 Four), Korea Advanced Institute of Science and Technology (KAIST), 291 Daehak-ro, Yuseong-gu, Daejeon 34141, Republic of Korea

[2]Green Carbon Research Center, Chemical Process Division, Korea Research Institute of Chemical Technology, 141 Gajeong-ro, Yuseong-gu, Daejeon 34114, Republic of Korea

[3]KAIST Institute for NanoCentury, KAIST, 291 Daehak-ro, Yuseong-gu, Daejeon 34141, Republic of Korea

[†]These authors contributed equally to this work.

*Correspondence: dongyeunkoh@kaist.ac.kr, sgim@kaist.ac.kr

Table of contents

Supplementary Fig. 1. Schematic illustration, and optical image and chemical structure.

Supplementary Fig. 2. FT-IR, GLP analysis and methanol permeance.

Supplementary Fig. 3. Deconvolution of high-resolution XPS spectra.

Supplementary Fig. 4. Surface morphology of XP membranes.

Supplementary Fig. 5. Cross-section SEM image of the PAN membrane.

Supplementary Fig. 6. Surface morphology of pV4D4/XP membranes.

Supplementary Fig. 7. Cross-sectional SEM image and elemental mapping images of 29nm/XP12 membrane.

Supplementary Fig. 8. ToF-SIMS depth profile spectra and 3D tomography.

Supplementary Fig. 9. ToF-SIMS depth profile spectra of pV4D4/XP membranes 1.

Supplementary Fig. 10. ToF-SIMS depth profile spectra of pV4D4/XP membranes 2.

Supplementary Fig. 11. ToF-SIMS depth profile spectra of pV4D4/XP membranes 3.

Supplementary Fig. 12. ToF-SIMS depth profile of 29nm/XP12 and 29nm/XP12-D.

Supplementary Fig. 13. Deconvolution of high-resolution XPS spectra.

Supplementary Fig. 14. AFM 3D images.

Supplementary Fig. 15. pV4D4 cross-linking structure analysis.

Supplementary Fig. 16. XRR spectrum of 74.24nm-thick pV4D4 thin film on Si wafer and simulated curve.

Supplementary Fig. 17. Stress-strain curve of membrane tensile testing results.

Supplementary Fig. 18. Single gas permeance of XP6 and 55nm/XP6 membranes for five gases with respect to kinetic diameter.

Supplementary Fig. 19. Schematic illustration of 3-cell cross-flow system for organic solvent nanofiltration.

Supplementary Fig. 20. Solvent permeance and solvent property plot.

Supplementary Fig. 21. Pure methanol flux with a time of 29nm/XP12-D under the 30 bar.

Supplementary Fig. 22. Polystyrene rejection profile with permeation time.

Supplementary Table. 1. Calculated etch rate of each constitutional region in the pV4D4, XP6 and XP12 membranes.

Supplementary Table. 2. Elemental content of PAN, XP12, and pV4D4 surface analyzed by XPS.

Supplementary Table. 3. Bulk density of polymers in comparison with other silicone polymers.

Supplementary Table. 4. Gas permeance and ideal selectivity of He and N_2 for pV4D4/XP membranes with different thicknesses.

Supplementary Table. 5. Physicochemical properties of organic solvents.

Supplementary Table. 6. Polystyrene rejection of 29nm/XP12-D, 30nm/XP6-D and 35nm/XP12-D membranes.

Supplementary Table. 7. OSN performance comparison with the state-of-the-art membranes.

Materials and Methods

Materials

Polyacrylonitrile (PAN, MW: 230,000 g mol^{-1}, Goodfellow) were further dried in vacuum oven at 60 °C during overnight. 1,3-Dioxolane (99%), dimethyl sulfoxide (DMSO, ≥ 99.9%), hydrazine monohydrate (≥ 98%) and trifluoroacetic acid (TFA, ≥ 99.5% (HPLC grade)) were obtained from Alfa Aesar. Different molecular weights of styrene oligomers standard (PS162, 266,370 and PS750) were purchased from Shimadzu Scientific Korea. 1,3,5,7-Tetravinyl-1,3,5,7-tetramethylcyclotetrasiloxane (V4D4, 95%, Gelest), *tert*-butyl peroxide (TBPO, 98%, Sigma-Aldrich), isopropyl alcohol (IPA, 99.5%, Daejung), tetrahydrofuran (THF, 99.9% (HPLC grade), Samchun), water (H_2O, HPLC grade, Daejung), acetic acid solution (HPLC grade, Sigma) and organic solvents (methyl alcohol (MeOH, 99.9% (HPLC grade), Samchun), ethyl alcohol (EtOH, 99.9% (HPLC), Samchun), acetone (ACT, 99.7% (HPLC), Samchun), acetonitrile (ACN, 99.9% (HPLC), Samchun), dimethylformamide (DMF, 99.7% (HPLC), Samchun)) were used as received without any purification.

Fourier transform infrared spectroscopy (FT-IR)

Fourier transform infrared (FT-IR) spectra were obtained in absorbance mode using an ALPHA FT-IR (Bruker Optics).

Gas liquid porometry (GLP)

Using IB-FT Germany Porolux™ 1000 Gas Liquid Porometry (GLP), the pore size distribution of the support membrane was measured. Two flow curves: one for the dry state and one for the wet state, were analyzed by GLP. The wet state was examined using a Porefil® liquid with a high surface tension (16 mN m^{-1}).

Scanning electron microscopy (SEM)

A Scanning Electron Microscope (Magellan 400, FEI company) was used to record the geometry and morphology of the support and composite membrane under 5-10 kV acceleration voltage, 50 pA-0.64 mA emission current and short working distance (4 mm or less). Membrane samples were soaked in liquid nitrogen to obtain intact morphology in the cross-sectional area. Then the samples were sputtered with platinum.

Atomic force microscopy (AFM)

The atomic force microscopy (AFM) topological images were obtained using a scanning probe microscope (XE-100, Park Systems) at a scan size of 5 μm x 5 μm.

X-ray photoelectron spectroscopy (XPS)

Chemical composition of the membrane, survey scan and depth profiling for each membrane was recorded with In-Situ X-ray photoelectron spectroscopy (Axis-Supra, Kratos).

X-ray reflectometry (XRR)

The XRR spectrum was obtained using a Rigaku SmartLab X-ray diffractometer using X-ray a wavelength of 1.541 Å. The bulk density of the pV4D4 film deposited on Si wafer was estimated by fitting XRR measurement and simulation values, executed using the GlobalFit software.

Grazing incidence wide angle X-ray scattering (GIWAXS)

Grazing incidence wide angle X-ray scattering (GIWAXS) measurement was performed on beamline 9 A(U-SAXS) in Pohang Accelerator Laboratory (Pohang, Korea) with E_k = 11.07 keV and a wavelength of 1.101 Å. For the structural properties of pV4D4, we deposited

the pV4D4 layer on a silicon substrate. The beam incidence angle was set in the range 0.12–0.15° which is between the critical angle of the pV4D4 film and the silicon substrate. The scattered beams from thin film were recorded by a two-dimensional CCD detector with a sample-to-detector distance of 0.22 m, and a GIWAXS pattern was obtained with the intensity for the scattering vector (q) in each direction.

Time-of-flight secondary ion mass spectrometry (ToF-SIMS)

Time-of-flight secondary ion mass spectrometry (ToF-SIMS, ION-TOF GmbH) was utilized to ascertain the structure of the pV4D4 composite membrane. Positive ion spectra and images were recorded with a 30 keV Bi_3^+ primary ions and using flood gun. SIMS analysis were performed 30 scanned 100 μm x 100 μm analysis area with 128 x 128 pixels. The $SiCH_3^+$ and $C_3H_4N^+$ ions were chosen to indicate each layer and depth profile were acquired using calculated etch rate in each region. The etch rate for the percolated region was estimated by the equation below:

$$\nu_{PR} = \frac{I_{\mathrm{PR}} - I_{\mathrm{XP}}}{I_{\mathrm{pV4D4}} - I_{\mathrm{XP}}} \times \nu_{\mathrm{pV4D4}} + \left(1 - \frac{I_{\mathrm{PR}} - I_{\mathrm{XP}}}{I_{\mathrm{pV4D4}} - I_{\mathrm{XP}}}\right) \times \nu_{\mathrm{XP}} \qquad (1)$$

where ν_{PR} is the etch rate of the V4D4-percolated region (Zone I) in the XP membrane, ν_{pV4D4} is the etch rate of the pV4D4 layer region (Zone II), ν_{XP} is the etch rate of the innate XP membrane region (Zone III), I_{PR} is the intensity of $SiCH_3^+$ in the V4D4-percolated region , I_{XP} is the mean intensity of $SiCH_3^+$ in the innate XP membrane region and I_{pV4D4} is the mean intensity of $SiCH_3^+$ in the pV4D4 layer region.

Optical property analysis

The refractive index and thickness of thin film were obtained by a spectroscopic ellipsometer (M2000, J. A. Woollam, USA). All ellipsometry profiles were fit to estimate refractive index and film thickness using the most suitable Cauchy model.

Single gas permeation

Single gas permeation tests were carried out with He, CO_2, O_2, N_2, and CH_4 at 35 °C under 1 bar of upstream pressure using the constant volume method. The downstream was kept in a vacuum state before the measurement started. When the feed is provided from the upstream and passes the membrane, the pressure transducer detects the pressure rise in the downstream volume. The gas permeance (GPU, 1 GPU = 10^{-6} cm^3 (STP) cm^{-2} s^{-1} cm Hg^{-1}) was evaluated from the pressure rise rate. The constant pressure method was also conducted with He and N_2 for estimating the membrane integrity under 1 - 3 bar feed pressure at 35 °C.

Supplementary Text

In the XRR analysis, the bulk density of thin film can be estimated by determining the critical angle, θ_c, for total reflection.[1] The X-ray refractive index of a material is represented in a complex number (Eq. 1) and is slightly less than 1 as a result of X-ray surface scattering and adsorption, where δ depends on the wavelength-dependent scattering associated with density and composition of the material (Eq. 2) and β is influenced by the X-ray adsorption (Eq. 3). The value of δ is on the order of 10^{-6} for X-ray with a wavelength of approximately ~ 1 Å, but that of β is nearly negligible, on the order of 10^{-8}.

$$n = 1 - \delta - i\beta \quad (1)$$

$$\delta = \left(\frac{r_e\lambda^2}{2\pi}\right) N_0\rho \sum_i x_i \left(z_i + f_i^{'}\right) / \sum_i x_i M_i \quad (2)$$

$$\beta = \left(\frac{r_e\lambda^2}{2\pi}\right) N_0\rho \sum_i x_i \left(z_i + f_i^{''}\right) / \sum_i x_i M_i \quad (3)$$

r_e: Classical radius of an electron (2.818 x 10^{-9} m)

N_0: Avogadro number

λ: X-ray wavelength

ρ: density (g cm^{-3})

z_i: Atomic number of the ith atom

M_i: Atomic weight of the ith atom

x_i: Atomic ratio (molar ratio) of the ith atom

f_i', f_i'': Atomic scattering factors of the ith atom (anomalous dispersion term)

When the incident angle of X-ray is tilted to the critical angle in an initial state where the X-ray beam is nearly parallel to the film surface, total reflection will occur. Since the refraction angle is 90 ° at the total reflection, Eq. 1 can be re-expressed as Eq. 4 using Snell's Law. As shown in Eq. 5, the monitored θ_c of the thin film depends on the δ value including the density information of the substance. This allows for the estimation of bulk density through the obtained θ_c for total reflection.

$$n \approx 1 - \delta\ ,\ 1 - \delta = \cos\theta_c \approx 1 - \frac{{\theta_c}^2}{2} \quad (4)$$

$$\theta_c \approx \sqrt{2\delta} \quad (5)$$

In our work, the θ_c for the pV4D4 thin film is 0.37 °, and the eventual bulk density is estimated to be 1.70 g cm^{-3}.

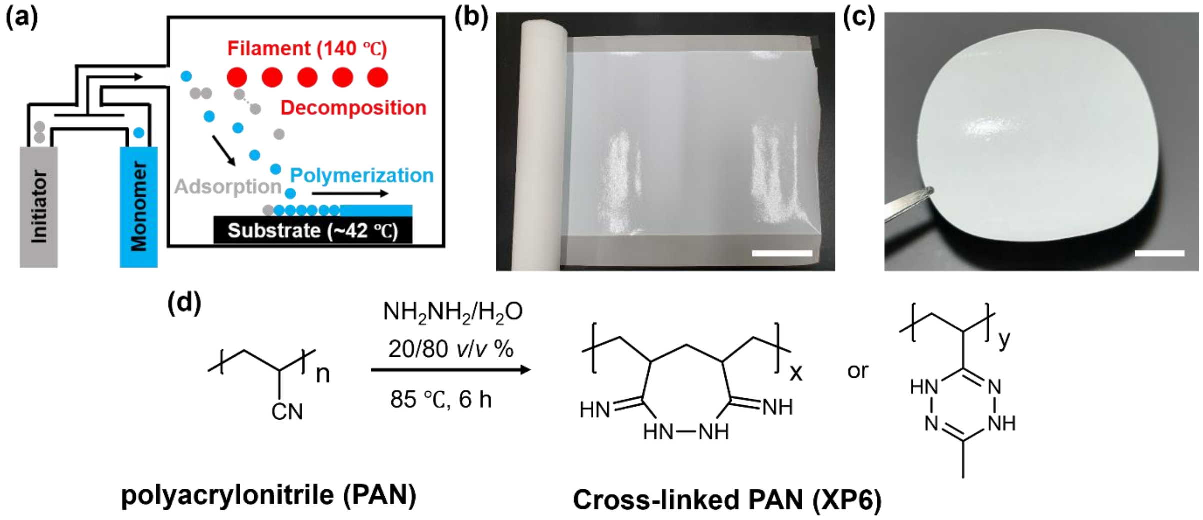

Supplementary Fig. 1. Schematic illustration, and optical image and chemical structure. (a) iCVD process. (b-c) Optical images of polyacrylonitrile (PAN) membrane and 6-hours cross-linked PAN (XP6) membrane. (d) Chemical reaction scheme of the PAN membrane cross-linked with a 20 *v*/*v*% hydrazine solution. Scale bar: 10 cm (b), 1 cm (c).

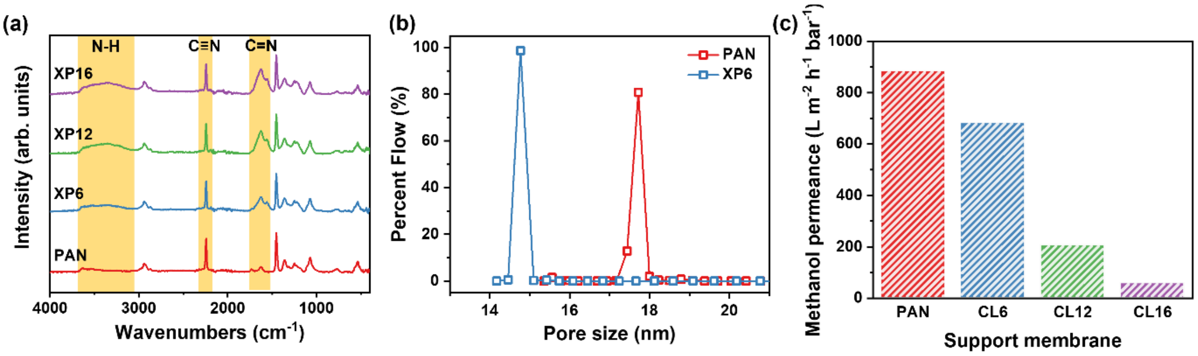

Supplementary Fig. 2. FT-IR, GLP analysis and methanol permeance. (a) FT-IR spectra of the support membranes with different cross-linking time. (b) Pore size distribution of pristine PAN and XP6 membranes. (c) Pure methanol permeance of support membranes.

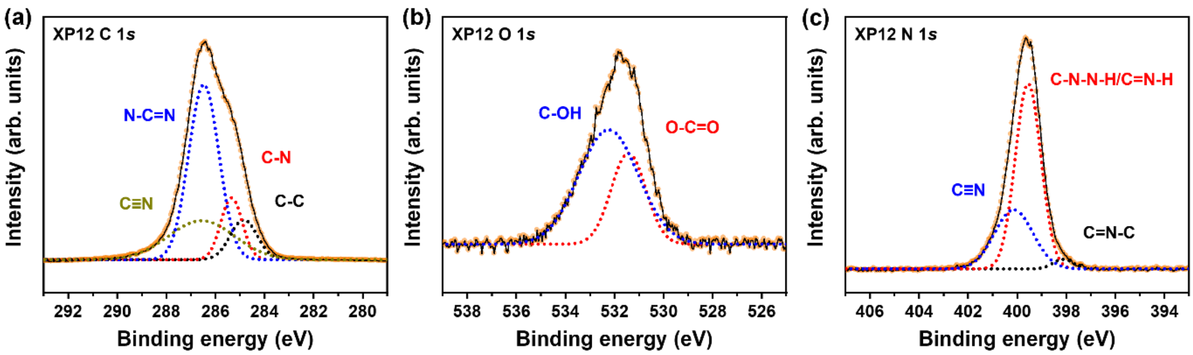

Supplementary Fig. 3. Deconvolution of high-resolution XPS spectra. (a-c) High-resolution XPS spectra of C 1*s*, O 1*s* and N 1*s* of XP12 membrane.

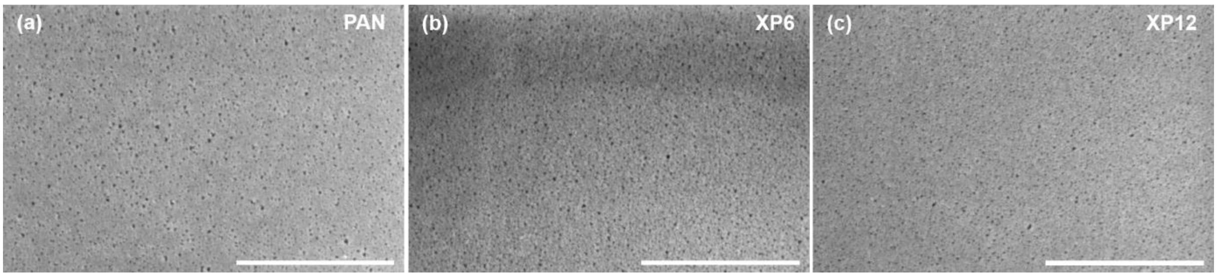

Supplementary Fig. 4. Surface morphology of XP membranes. (a-c) Top-view SEM images of PAN, XP6 and XP12 membranes. Scale bar: 1 µm

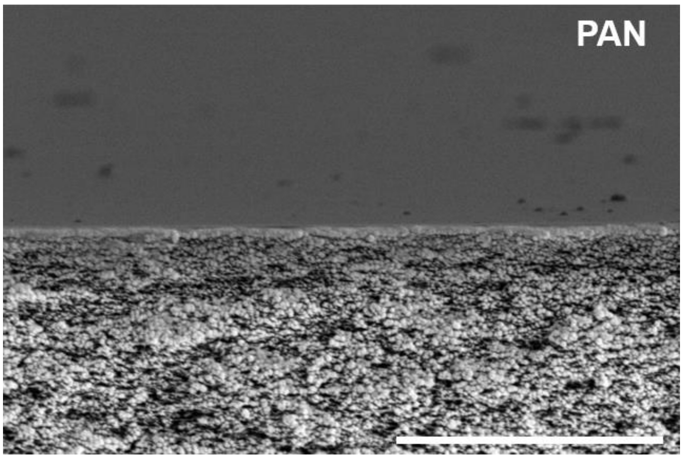

Supplementary Fig. 5. Cross-section SEM image of the PAN membrane. Scale bar: 5 µm

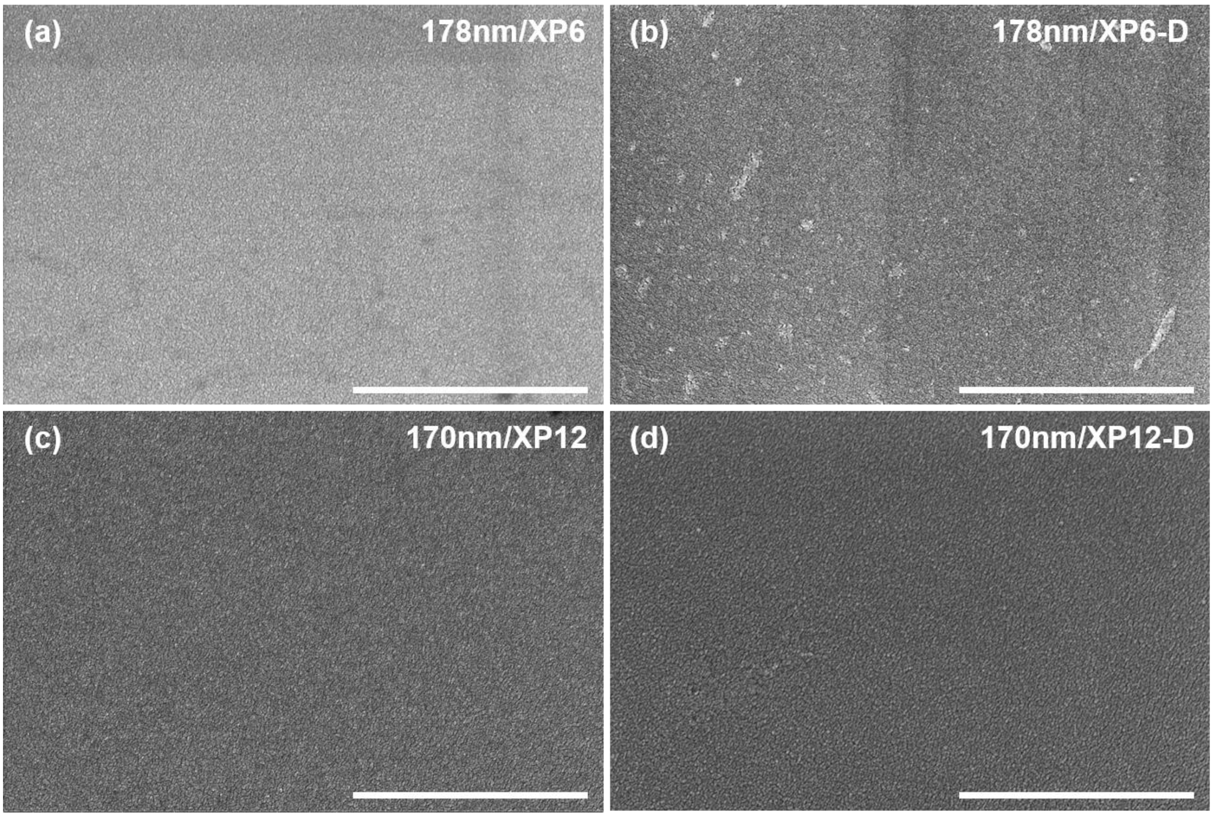

Supplementary Fig. 6. Surface morphology of pV4D4/XP membranes. (a-d) Surface SEM images of 178nm/XP6, 178nm/XP6-D, 170nm/XP12 and 170nm/XP12-D membranes. Scale bar: 1 µm

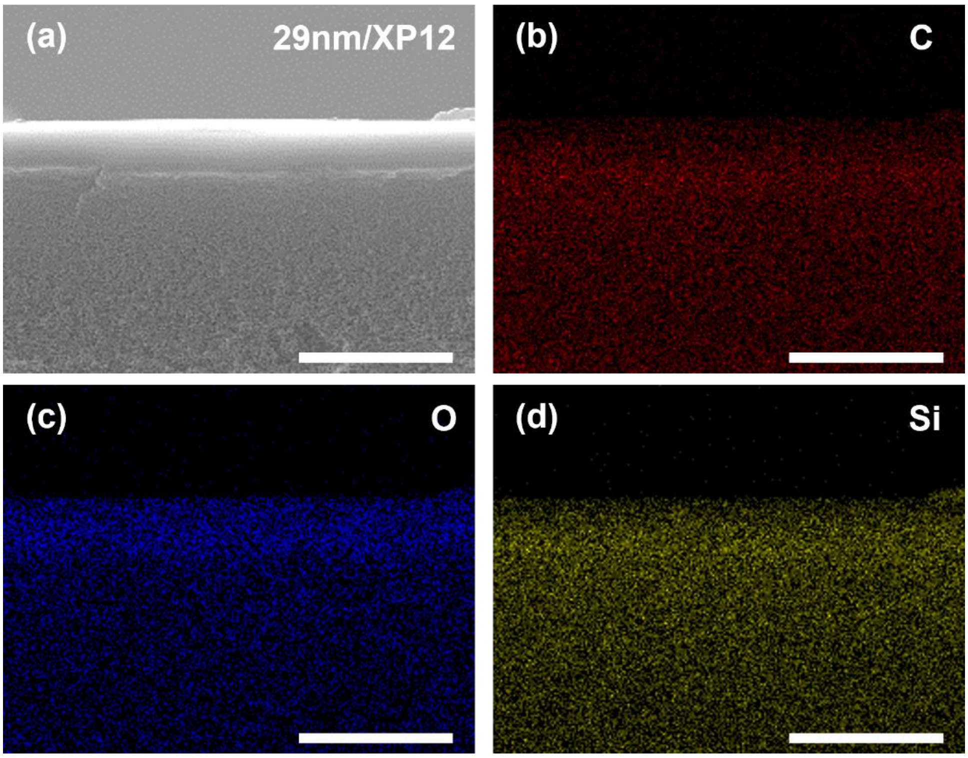

Supplementary Fig. 7. Cross-sectional SEM image and elemental mapping images of 29nm/XP12 membrane. (a) Cross-sectional SEM image of the 29nm/XP12 membrane, along with (b-d) elemental mapping images of carbon, oxygen, and silicon, respectively. Scale bar: 3 μm

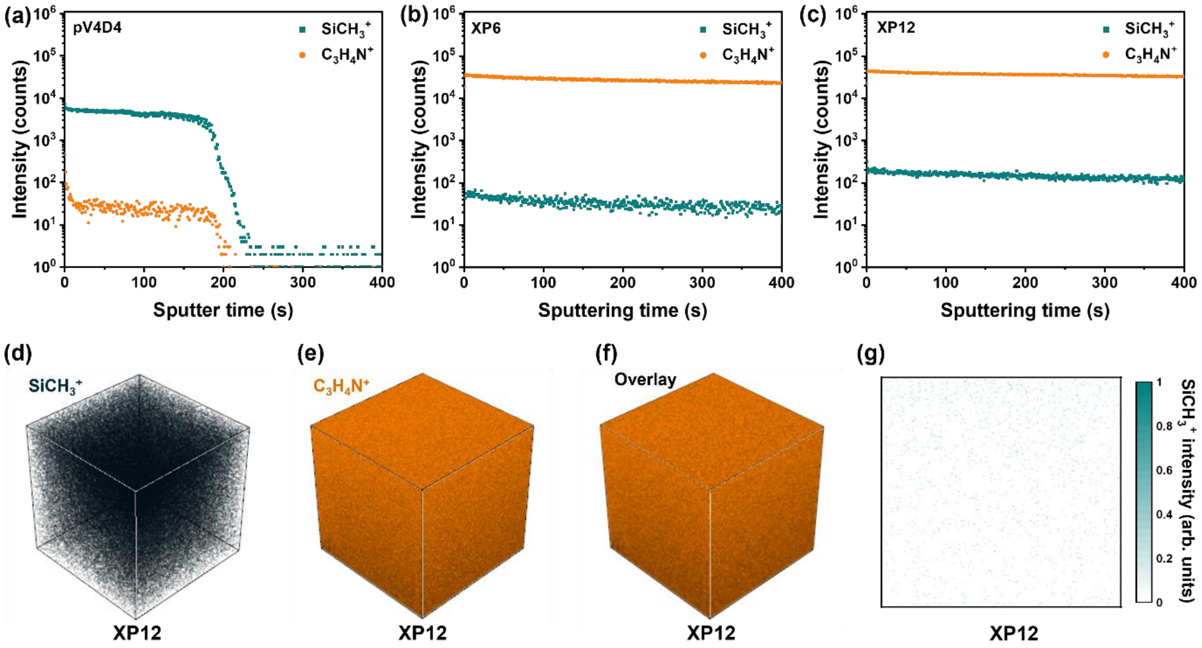

Supplementary Fig. 8. ToF-SIMS depth profile spectra and 3D tomography. (a-c) ToF-SIMS depth profile spectra of pV4D4 polymer layer on Si wafer, XP6 and XP12 membranes. (d-f) 3D images of $SiCH_3^+$, $C_3H_4N^+$ ion and overlay image in XP12. (g) 2D spectra of $SiCH_3^+$ ion in XP12. 3D tomography size: 100 μm x 100 μm x 10 μm.

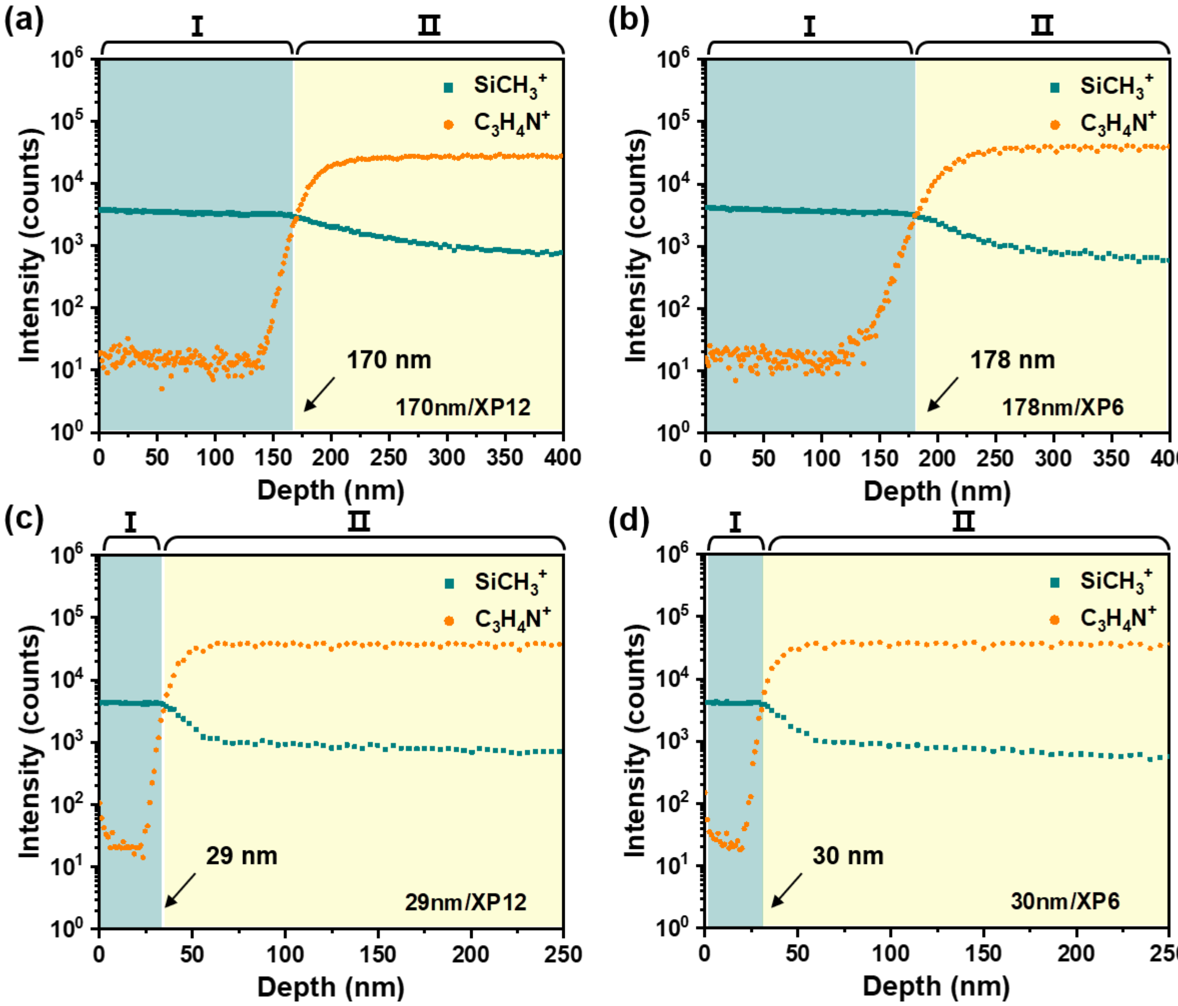

Supplementary Fig. 9. ToF-SIMS depth profile spectra of pV4D4/XP membranes 1. (a-d) ToF-SIMS depth profile spectra of 170nm/XP12, 178nm/XP6, 29nm/XP12 and 30nm/XP6.

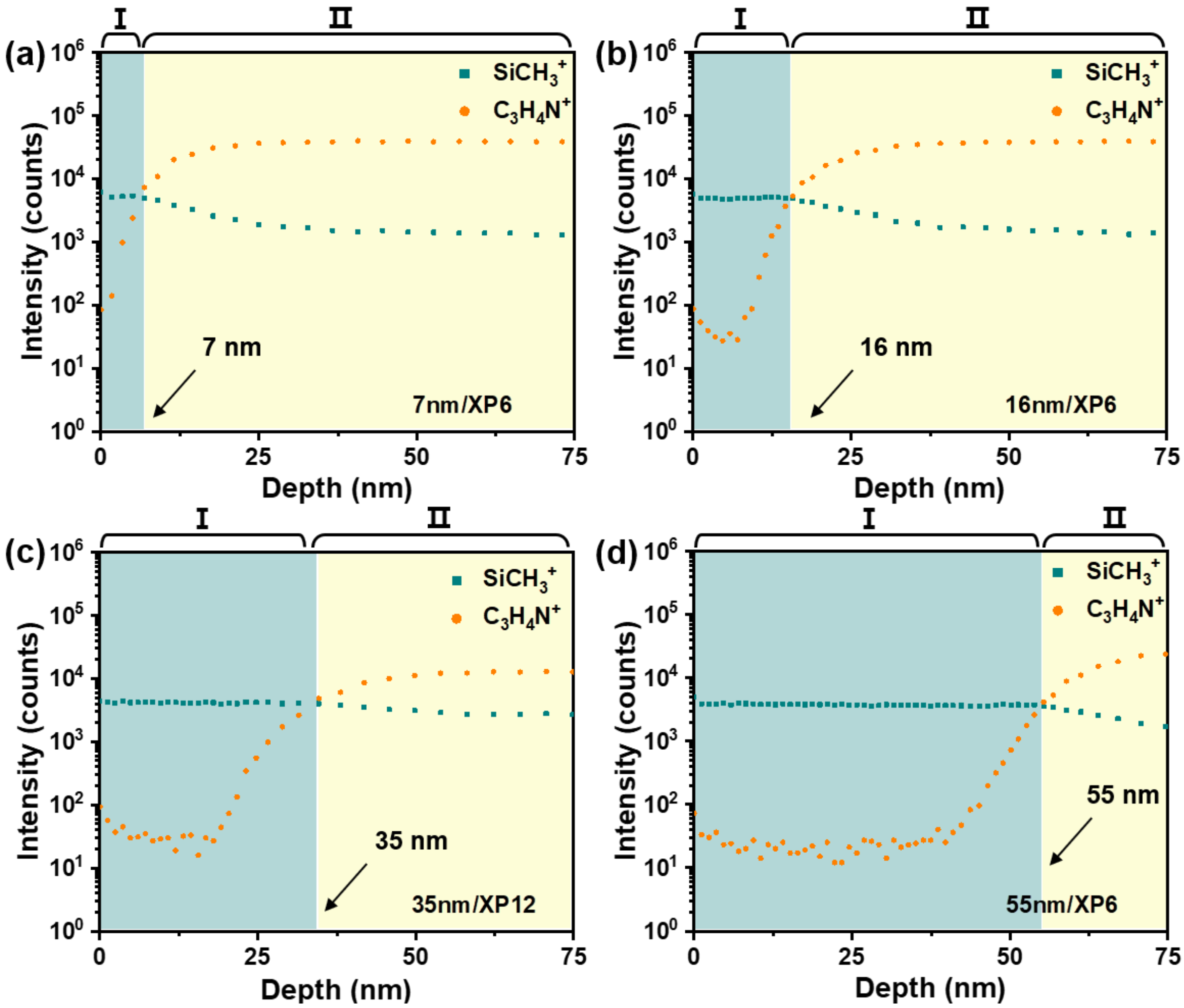

Supplementary Fig. 10. ToF-SIMS depth profile spectra of pV4D4/XP membranes 2. (a-d) ToF-SIMS depth profile spectra of 7nm/XP6, 16nm/XP6, 35nm/XP12 and 55nm/XP6.

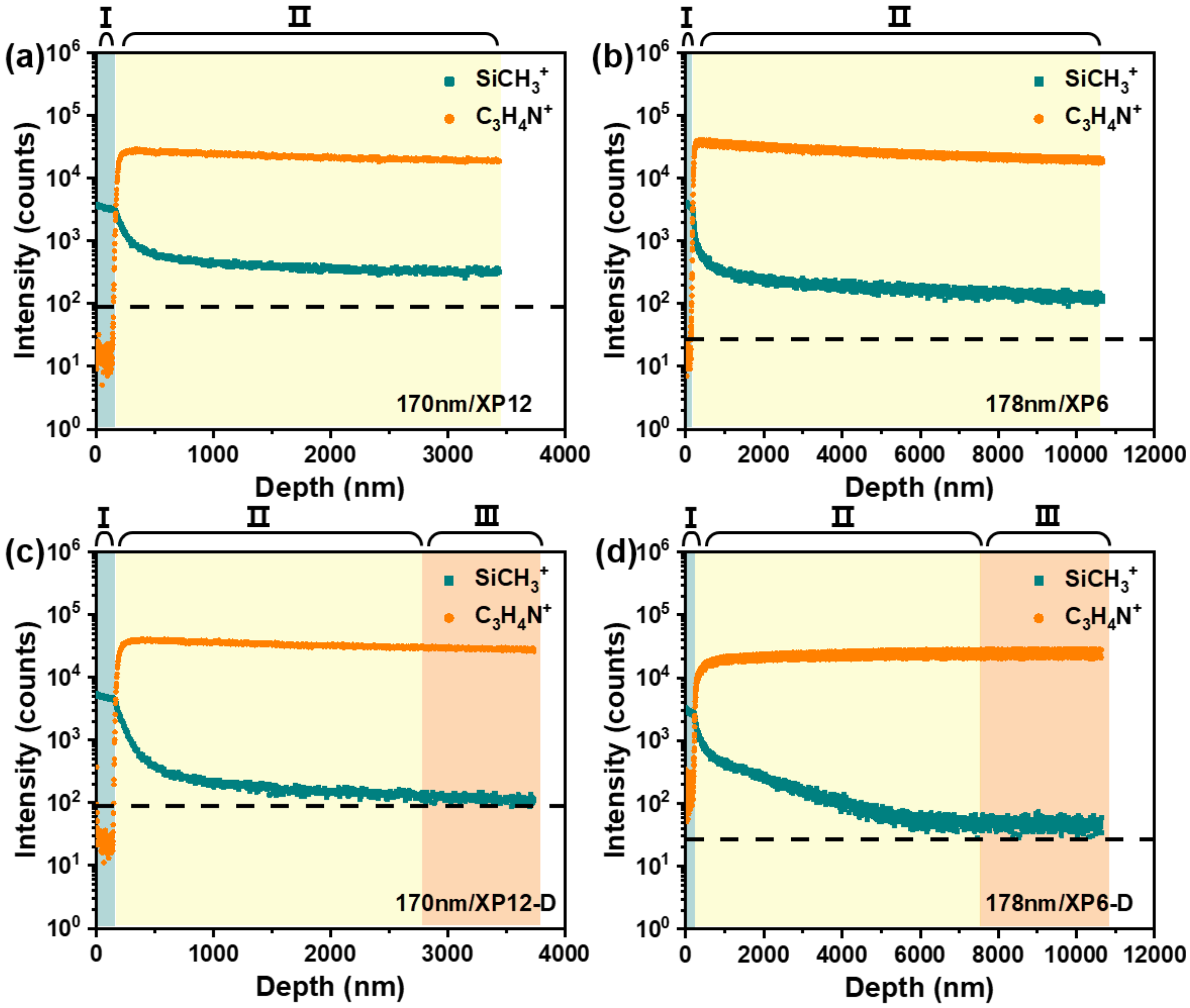

Supplementary Fig. 11. ToF-SIMS depth profile spectra of pV4D4/XP membranes 3. (a-d) ToF-SIMS depth profile spectra of 170nm/XP12, 178nm/XP6, 170nm/XP12-D and 178nm/XP6-D.

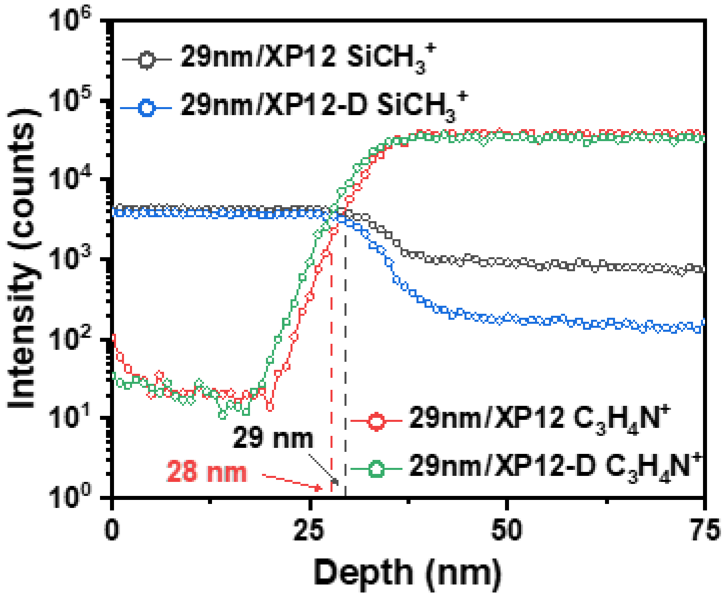

Supplementary Fig. 12. ToF-SIMS depth profile of 29nm/XP12 and 29nm/XP12-D. $SiCH_3^+$ and $C_3H_4N^+$ ion intensities of 29nm/XP12 and 29nm/XP12-D.

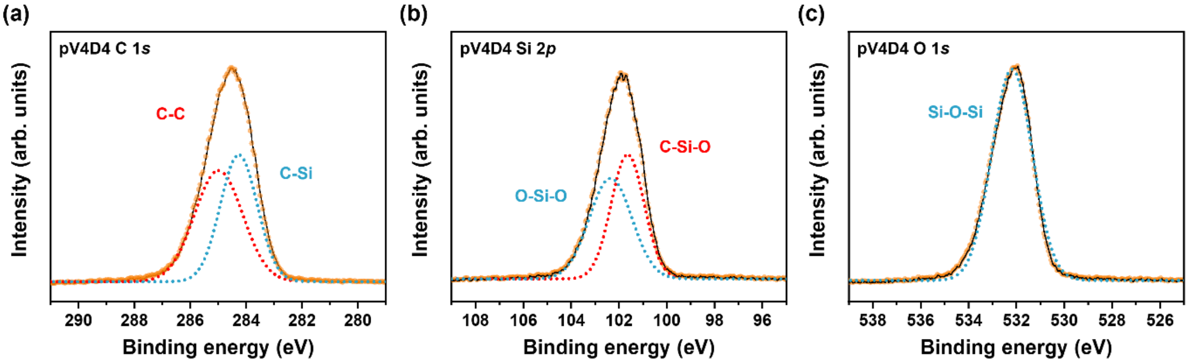

Supplementary Fig. 13. Deconvolution of high-resolution XPS spectra. (a-c) C 1*s*, Si 2*p* and O 1*s* high-resolution XPS spectra of the pV4D4 polymer on Si wafer.

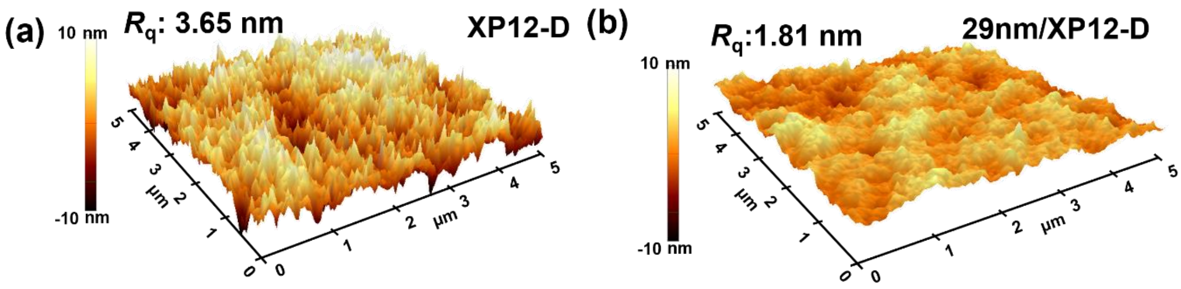

Supplementary Fig. 14. AFM 3D images. (a-b) AFM images of XP12 and 29nm/XP12 membrane after soaking in DMF for 24 hours.

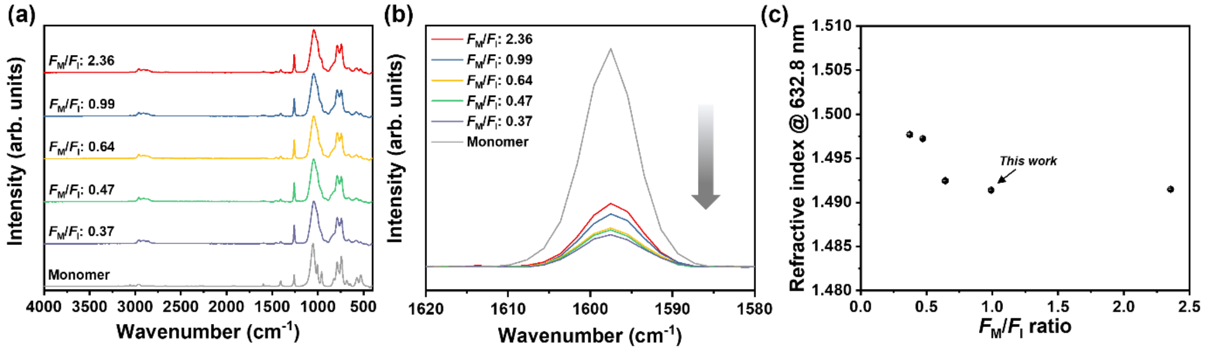

Supplementary Fig. 15. pV4D4 cross-linking structure analysis. (a-b) FT-IR spectra and (c) refractive index of pV4D4 films with varying monomer-to-initiator ratios.

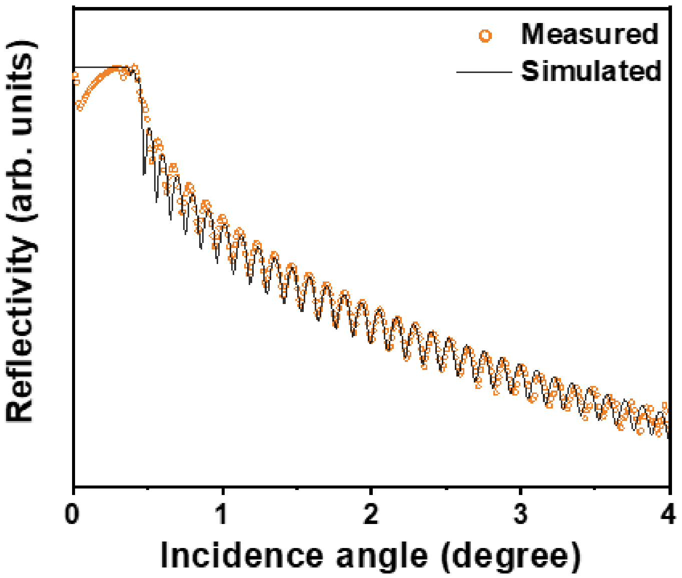

Supplementary Fig. 16. XRR spectrum of 74.24nm-thick pV4D4 thin film on Si wafer and simulated curve.

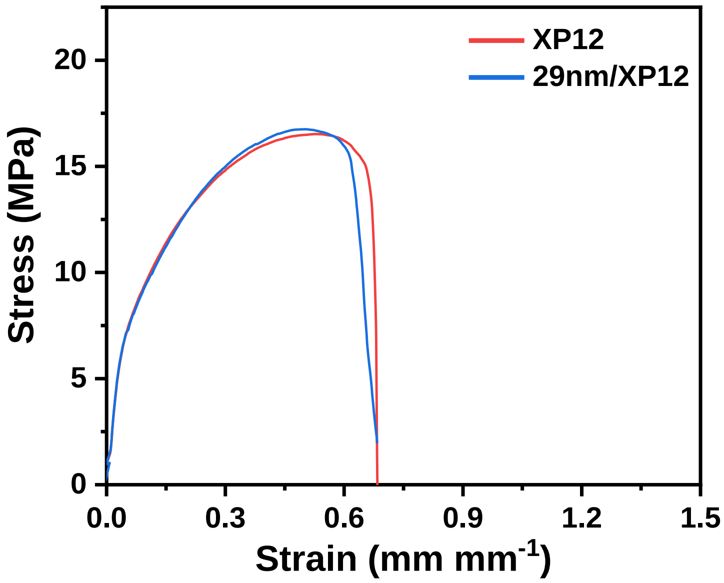

Supplementary Fig. 17. Stress-strain curve of membrane tensile testing results. All tensile test was performed using a universal testing machine (5 kN AllRound table top, ZwickRoell, Germany). The film specimens were prepared with the following dimensions: 10 mm in width, 50 mm in length, and 220 μm in thickness. The film membranes were pulled at strain rate of 20 mm min^{-1} at room temperature, starting from the initial grip gap of 10 mm.

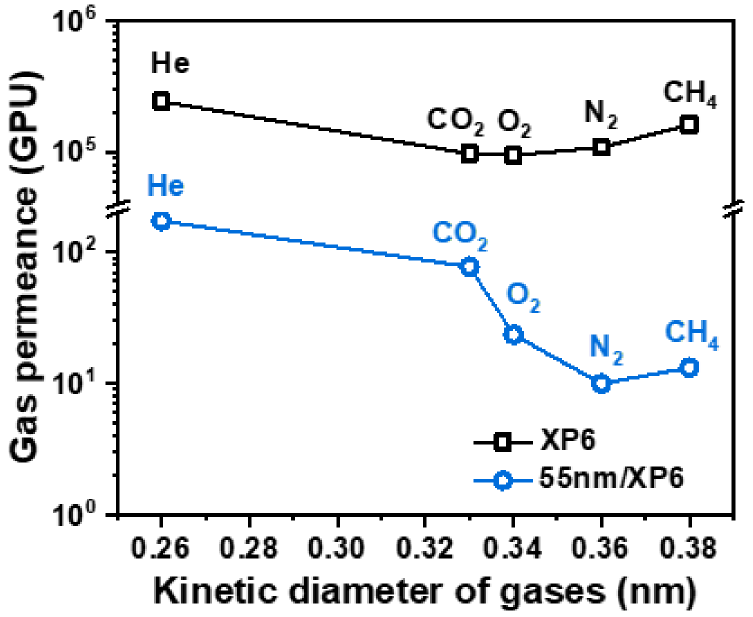

Supplementary Fig. 18. Single gas permeance of XP6 and 55nm/XP6 membranes for five gases with respect to kinetic diameter.

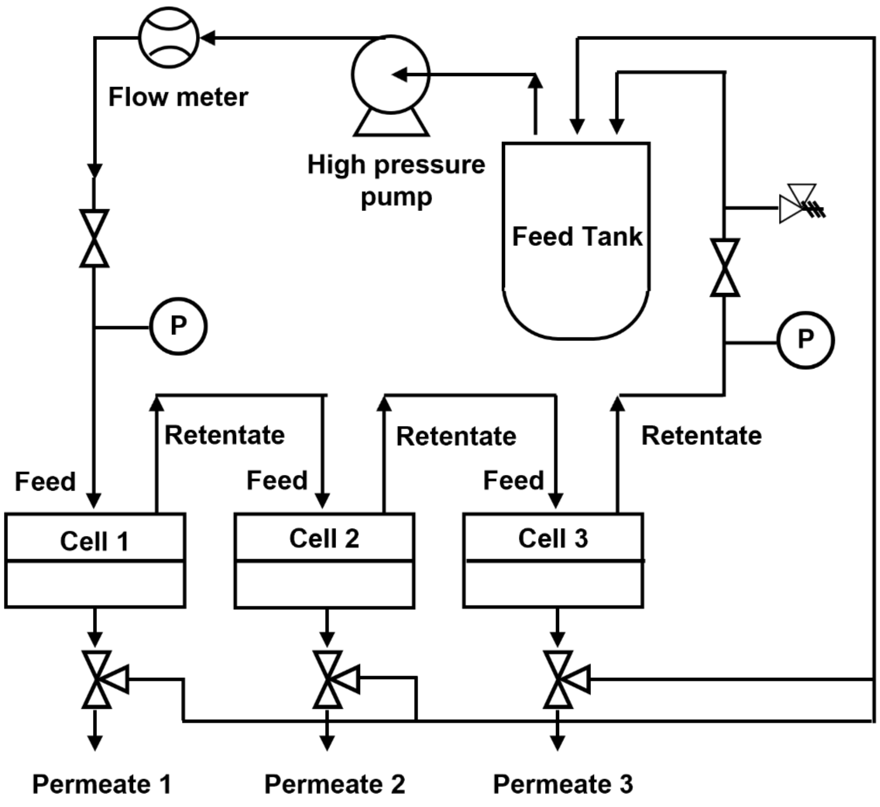

Supplementary Fig. 19. Schematic illustration of 3-cell cross-flow system for organic solvent nanofiltration.

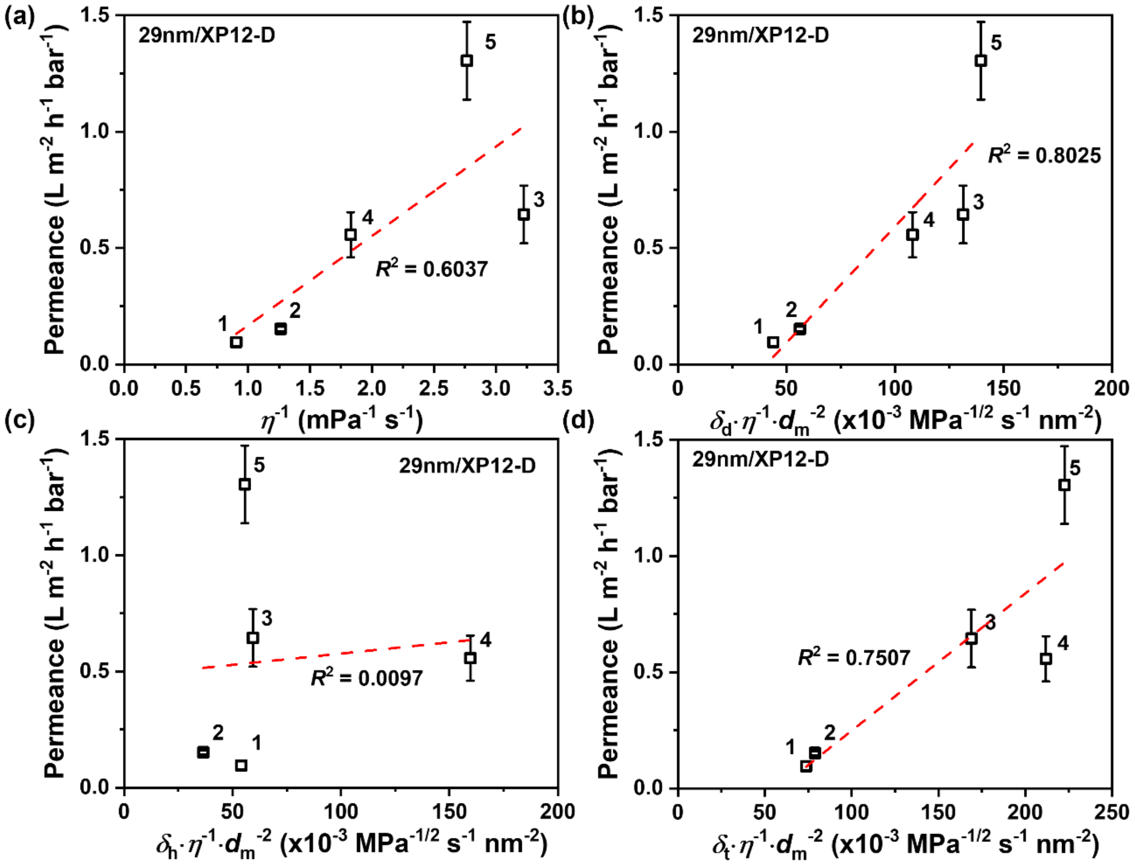

Supplementary Fig. 20. Solvent permeance and solvent property plot. Poor correlation between the solvent permeance of 29nm/XP12-D membrane and (a) inverse of viscosity (η^{-1}) or other combined solvent property with different solubility parameters related to (b) dispersion (δ_d), (c) hydrogen bond (δ_h) and (d) total (δ_t). 1: ethanol, 2: dimethylformamide, 3: acetone, 4: methanol, 5: acetonitrile. All error bars indicate the standard deviation at two or three measurements.

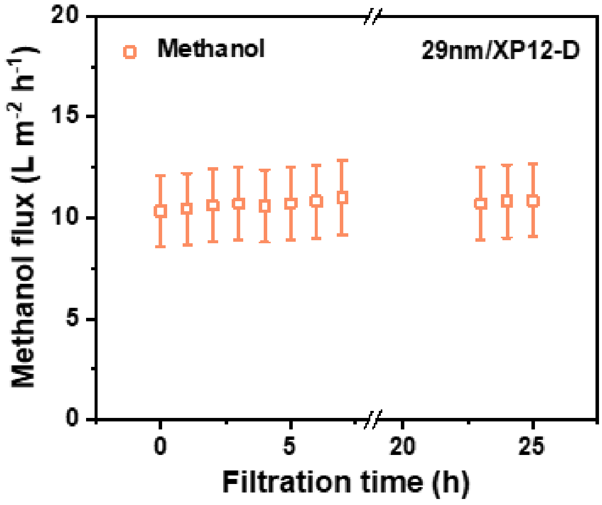

Supplementary Fig. 21. Pure methanol flux with a time of 29nm/XP12-D under the 30 bar. All error bars indicate the standard deviation at two or three measurements.

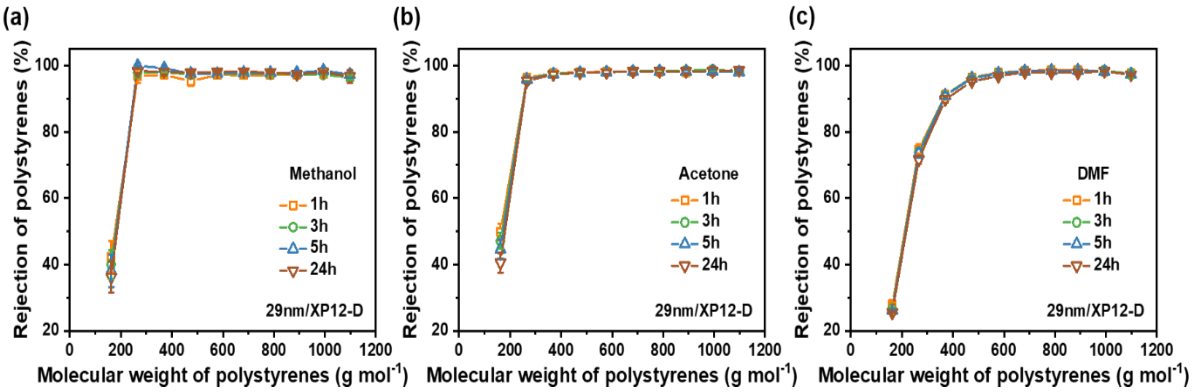

Supplementary Fig. 22. Polystyrene rejection profile with permeation time. (a-c) Polystyrene rejection profile of 29nm/XP12-D under methanol, acetone and DMF solvents over time. All error bars indicate the standard deviation at two or three measurements.

Supplementary Table 1. Calculated etch rate of each constitutional region in the pV4D4, XP6 and XP12 membranes.

Region	Etch rate (nm/s)
pV4D4	1.24
XP12	4.83
XP6	5.99

Supplementary Table 2. Elemental content of PAN, XP12, and pV4D4 surface analyzed by XPS.

Membrane	Elemental ratio (relative atom, %)				
	C	O	N	Si	N/C ratio
PAN	75.4	2.7	21.9	-	0.29
XP12	73.3	4.4	22.3	-	0.30
pV4D4	57.5	22.2	-	20.3	-

Supplementary Table 3. Bulk density of polymers in comparison with other silicone polymers.

Silicone polymer	Density (g cm^{-3})	Reference
PDMS*	0.97	2
BTESE*	1.50	3
pV4D4	1.70	**This work**

* Abbreviations: PDMS-poly(dimethylsiloxane), BTESE-1,2-bis(triethoxysilyl)ethane

Supplementary Table 4. Gas permeance and ideal selectivity of He and N_2 for pV4D4/XP membranes with different thicknesses.

Membrane	He permeance (GPU)	N_2 permeance (GPU)	He/N_2 ideal selectivity
7nm/XP6	26928	10008	2.69
16nm/XP6	235.05	25.83	9.10
55nm/XP6	173.65	9.98	17.88
35nm/XP12	178.41	14.45	12.35
35nm/XP12-D	282.06	44.23	6.38
Knudsen diffusion	-	-	2.65

Supplementary Table 5. Physicochemical properties of organic solvents.

Solvents	Molar diameter, d_m (nm)	Viscosity, η (mPa·s)	Hansen solubility parameter (MPa$^{1/2}$)			
			δ_d	δ_p	δ_h	$\delta=\sqrt{\delta_d^2+\delta_p^2+\delta_h^2}$
MeOH	0.51	0.54	15.1	12.3	22.3	29.7
EtOH	0.57	1.08	15.8	8.8	19.4	26.6
ACT	0.62	0.32	15.5	10.4	7.0	20.1
ACN	0.55	0.34	15.3	18.0	6.1	24.6
DMF	0.63	0.82	17.4	13.7	11.3	24.8

Supplementary Table 6. Polystyrene rejection of 29nm/XP12-D, 30nm/XP6-D and 35nm/XP12-D membranes.

Membrane (Solvent)	Solvent permeance (L m^{-2} h^{-1} bar^{-1})	Polystyrene rejection (%)									
		162 g mol^{-1}	266 g mol^{-1}	370 g mol^{-1}	474 g mol^{-1}	578 g mol^{-1}	682 g mol^{-1}	786 g mol^{-1}	890 g mol^{-1}	994 g mol^{-1}	1098 g mol^{-1}
29nm/XP12-D (MeOH)	0.225 ± 0.043	36.24 ± 4.74	98.40 ± 0.70	98.07 ± 0.79	97.89 ± 0.34	98.06 ± 0.35	98.16 ± 0.31	97.94 ± 0.26	97.35 ± 0.40	97.99 ± 0.36	96.71 ± 1.88
29nm/XP12-D (ACT)	0.644 ± 0.124	40.51 ± 2.98	95.41 ± 1.01	97.31 ± 0.77	97.86 ± 0.69	97.97 ± 0.67	98.27 ± 0.54	98.29 ± 0.67	98.25 ± 0.75	98.29 ± 1.18	98.62 ± 0.58
29nm/XP12-D (DMF)	0.152 ± 0.008	25.44 ± 0.18	71.61 ± 1.29	89.77 ± 0.96	95.20 ± 0.46	96.81 ± 0.35	97.96 ± 0.23	97.85 ± 0.26	97.83 ± 0.40	98.29 ± 0.08	97.20 ± 0.37
30nm/XP6-D (MeOH)	0.303 ± 0.013	27.97 ± 9.74	61.46 ± 3.97	69.61 ± 3.87	75.06 ± 5.02	78.41 ± 4.88	80.87 ± 4.71	82.48 ± 4.68	84.43 ± 3.88	85.13 ± 4.59	84.23 ± 5.20
30nm/XP6-D (ACT)	0.715 ± 0.024	41.70 ± 3.19	59.15 ± 2.78	65.92 ± 1.91	65.42 ± 1.05	69.08 ± 0.81	72.25 ± 0.45	74.40 ± 0.46	76.73 ± 0.72	79.13 ± 0.77	81.36 ± 0.31
30nm/XP6-D (DMF)	0.100 ± 0.005	26.48 ± 4.02	56.48 ± 11.81	68.61 ± 11.97	76.10 ± 9.78	79.54 ± 8.89	82.11 ± 8.14	84.03 ± 7.30	86.21 ± 7.23	88.10 ± 5.45	88.99 ± 8.48
35nm/XP12-D (MeOH)	0.131 ± 0.006	82.1 ± 5.51	98.21 ± 0.05	98.64 ± 0.00	98.60 ± 0.03	98.61 ± 0.03	98.70 ± 0.04	98.79 ± 0.08	98.66 ± 0.15	98.18 ± 0.22	97.61 ± 0.45
35nm/XP12-D (ACT)	0.444 ± 0.111	85.16 ± 5.69	97.81 ± 0.76	98.34 ± 0.56	98.49 ± 0.40	98.56 ± 0.29	98.76 ± 0.26	98.82 ± 0.45	98.74 ± 0.43	98.87 ± 0.32	98.55 ± 0.73
35nm/XP12-D (DMF)	0.089 ± 0.003	78.84 ± 0.39	90.66 ± 1.18	92.71 ± 1.61	94.71 ± 1.27	95.54 ± 1.59	95.93 ± 1.68	96.12 ± 0.72	97.23 ± 0.50	98.51 ± 0.08	98.67 ± 0.52

Supplementary Table 7. OSN performance comparison with the state-of-the-art membranes.

Membrane material	Methanol permeance (L m^{-2} h^{-1} bar^{-1})	MW: 150-250 g mol^{-1}		MW: 250-350 g mol^{-1}		Selectivity	Ref.
		Marker (MW)	Rejection (%)	Marker (MW)	Rejection (%)	MW:150-250/ MW 250-350	
Aligned macrocycle	7.64	AZB (182)	33.9	MO (327)	93	9.44	4
PIM	8.7	SOG (246)	99.3	DR (314)	99.9	7.00	5
Polyamide	13.2	HNSA (246)	89.6	MO (327)	96	2.60	6
Polyarylate	8	CSG (249)	70	DR (314)	90	3.00	7
Graphene oxide	9	HNSA (246)	99.9	DR (314)	99.9	1.00	8
Conjugated polymers	22.5	AZB (182)	10.2	MB (320)	48.4	1.74	9
COF	72	NR (229)	2	SO (351)	5	1.03	10
**29nm/XP12-D**	**0.225**	**PS oligomer (162)**	**36.2**	**PS oligomer (266)**	**98.4**	**39.88**	**This work**
**16nm/XP6**	**0.016**	**PS oligomer (162)**	**63.1**	**PS oligomer (266)**	**97.6**	**15.38**	**This work**

*The molecular weight (MW) of each marker was indicated in brackets following the marker name.
*Abbreviations: PIM-polymer of intrinsic microporosity and COF-covalent organic framework.

References

1. Yasaka, M. & Others. X-ray thin-film measurement techniques. *The Rigaku Journal* **26**, 1–9 (2010).
2. Yamada, Y., Ichii, T., Utsunomiya, T. & Sugimura, H. Visualizing polymeric liquid/solid interfaces by atomic force microscopy utilizing quartz tuning fork sensors. *Jpn. J. Appl. Phys. (2008)* **59**, SN1009 (2020).
3. Cui, L. *et al.* Atmospheric plasma deposited dense silica coatings on plastics. *ACS Appl. Mater. Interfaces* **4**, 6587–6598 (2012).
4. Jiang, Z. *et al.* Aligned macrocycle pores in ultrathin films for accurate molecular sieving. *Nature* **609**, 58–64 (2022).
5. Ali, Z. *et al.* Finely Tuned Submicroporous Thin-Film Molecular Sieve Membranes for Highly Efficient Fluid Separations. *Adv. Mater.* **32**, e2001132 (2020).
6. Lu, Y. *et al.* Polyamide Thin Films Grown on PD/SWCNT-Interlayered-PTFE Microfiltration Membranes for High-Permeance Organic Solvent Nanofiltration. *Ind. Eng. Chem. Res.* **59**, 22533–22540 (2020).
7. Jimenez-Solomon, M. F., Song, Q., Jelfs, K. E., Munoz-Ibanez, M. & Livingston, A. G. Polymer nanofilms with enhanced microporosity by interfacial polymerization. *Nat. Mater.* **15**, 760–767 (2016).
8. Yang, Q. *et al.* Ultrathin graphene-based membrane with precise molecular sieving and ultrafast solvent permeation. *Nat. Mater.* **16**, 1198–1202 (2017).
9. Liang, B. *et al.* Microporous membranes comprising conjugated polymers with rigid backbones enable ultrafast organic-solvent nanofiltration. *Nat. Chem.* **10**, 961–967 (2018).
10. Shinde, D. B. *et al.* Crystalline 2D Covalent Organic Framework Membranes for High-Flux Organic Solvent Nanofiltration. *J. Am. Chem. Soc.* **140**, 14342–14349 (2018).
